# Potential Global-Local Inconsistency in Species-Area Relationships Fitting

**DOI:** 10.3389/fpls.2016.01282

**Published:** 2016-08-30

**Authors:** Xubin Pan, Xiuling Zhang, Feng Wang, Shuifang Zhu

**Affiliations:** ^1^Institute of Plant Quarantine, Chinese Academy of Inspection and QuarantineBeijing, China; ^2^School of Mathematics, Tsinghua UniversityBeijing, China; ^3^Institute of Desertification Studies, Chinese Academy of ForestryBeijing, China

**Keywords:** log-normal distribution, negative-binomial distribution, power SAR, logarithm SAR, species-abundance distribution, extrapolation

## Abstract

The Species-Area Relationship (SAR) has been widely employed to assess species diversity and predict species extinction. Thus far, although many functions were proposed to fit SAR based on field observations or simulation results, the shape of SAR curve has been debated extensively over decades. Here we uncover a potential global-local inconsistency in SARs fitting simulation blocked by the limitation of large area sampling before. The results indicated that power and logarithm SAR formulas were good for the fitting if the sampling area range is not large which is also the practical sampling interval in the field. However, for the logarithm SAR fitting, a sigmoid curve occurred in the log_10_ Area−Number of Species plane, and for the power SAR fitting, the curve is convex instead of a straight line as assumed when linear regression was applied. In conclusion, neither the power SAR nor the logarithm SAR fitted to simulated data is linear at large sampling range as commonly assumed in previous studies, no matter the distribution of species abundance is log-normal or negative-binomial, which unmasks the global-local inconsistency in SARs fitting. Thus, misestimates of total number of species or other derivation parameters can occur if the fitted relationship is extrapolated beyond the range of the small and intermediate sampling size.

## Introduction

Species-Area Relationship (SAR) is one of the most studied patterns in ecology, and has been widely employed to assess species diversity and predict species extinction (Tjørve and Tjørve, 2008). Thus far, although many functions were proposed for fitting SAR based on field observations or simulation results, the shape of SAR curve has been debated extensively over decades (Tjørve, 2003; Tjørve et al., 2008). Among various functions of SAR, two are best known and most commonly applied, the power format proposed by Arrhenius (1921), $S_{A}=cA^{z}$, where *S*_*A*_ is the number of species in area *A*, and *c* and *z* are fitted constants (Arrhenius, 1921), and the logarithm format proposed by Gleason (1922), $S_{A}=a+b^{*}lnA$, where *a* and *b* are fitted constants (Gleason, 1922). Compared to logarithm SAR, power SAR has parameters corresponding to ecological meanings (Tjørve, 2003), where *c* is the number of species per area (analogous to α diversity) and *z* is the self-similarity index (analogous to β diversity) (Tjørve and Tjørve, 2008). The power SAR was even proposed as a universal model (Dengler, 2008). The application of the power SAR, however, is still in controversy due to potential risks in the process of sampling and parameters estimating, which often leads to underestimate or overestimate of species diversity and extinction rate (Collins et al., 2002; He and Hubbell, 2011; Pan, 2013, 2015). One reason is that an important global factor of the identification of total area and corresponding total number of species has been overlooked for years (Pan, 2013, 2015).

Moreover, the shape of SAR curve can be affected by species-abundance distribution (SAD), and several studies attempted to address potential links between the two (He and Legendre, 2002; Green and Ostling, 2003; Tjørve and Tjørve, 2008). For example, for the community with species distributed randomly and independently, SAR can be calculated from SAD (the formula is shown in Methods) (Coleman, 1981). Obviously, the way of sampling is crucial for bridging the SAR and SAD, and accurate fitting is possible only if complete and detail sampling is carried out in accordance with statistic requirement. However, since detail sampling at a large scale is not practical, the fitting (i.e., parameterization) of SAR is usually based on the sampling at a small scale. However, high goodness of fit at the local range does not necessarily expect the same goodness of fit at the global range, partly because local sampling is more likely to misestimate or overlook the existence of rare species (Preston, 1948; Verberk, 2011).

Compared to field sampling subject to incomplete surveying, computer simulation sampling can provide a more feasible approach to fitting SAR (Tjørve and Tjørve, 2008). Moreover, computer simulation enables us to scrutinize the patterns of SAR at any level, and thereby can help explore whether the inconsistency of SAR may occur between global and local levels.

As abovementioned, the range of sampling is crucial for fitting SAR, and therefore this study will try to reveal potential misguidance and risks of extrapolation. In this study, we tested whether the patterns of the two SARs were consistent at the global and local levels through numerical analysis. The power and logarithm SARs were used to simulate data from two types of species-abundant distributions (negative-binomial (NB) and log-normal (LN) distributions) at the global level. We also evaluated parameter variation and potential misguidance of extrapolation.

## Methods

### Data simulation

A simulation program in the R platform (R version 3.2.0, R Core Team, 2015) was used to generate sampling data. The total area was set as 1,000,000 points, and each individual of every species occupied one point. The occurrence of plant species was simulated following two distribution patterns, negative-binomial (NB) and log-normal (LN) distributions (selected from dozens of SADs, McGill et al., 2007). Individuals of 100 and 500 species were generated randomly at initial status of simulation species distribution.

### Data transformation

As former studies proposed (Coleman, 1981), for a community where resident species is distributed randomly and independently, the SAR curve can be formulated as R Core Team (2015)

(1) $$\begin{array}{l} S_{A}=S_{TA}-\sum_{i=1}^{S_{TA}}{\left(1-\frac{A}{TA}\right)}^{N_{i}}=\sum_{i=1}^{S_{TA}}\left(1-{\left(1-\frac{A}{TA}\right)}^{N_{i}}\right)\; \end{array}$$

where *S*_*TA*_ is the total number of species in the total area (*TA*), and *N*_*i*_ is the number of individuals of per species *i*. This formula was used to calculate SAR based on simulated data.

Two functions, which are $S_{A}=a+b^{*}lnA$ (i.e., the logarithm SAR) and log*S*_*A*_ = log*c* + *z*log*A* (i.e., the logarithm format of the power SAR) are used to fit SARs based on simulated data. Thus, the area is log-transformed in both fittings. The number of species was not transformed in the logarithm SAR fitting but log-transformed in the power SAR fitting.

## Results and discussion

**Figure 1:**
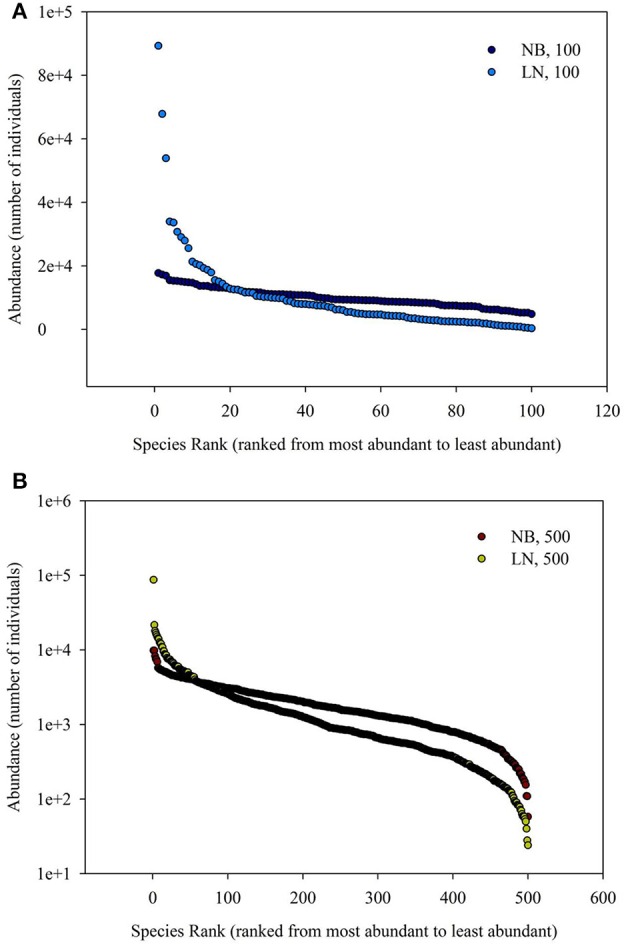
**(A)** Is the diagram for Species Rank (ranked from most abundant to least abundant)−Abundance (number of individuals). **(B)** Is the schematic diagram for log_10_ (Area)−log_10_ (Number of Species). Different SAD (negative-binomial, NB; log-normal, LN) and number of total species (100 and 500).

The simulated SADs are shown in Figure 1. The range of number of individuals of each species of negative-binomial distribution is smaller than that of log-normal distribution, which means the latter has more rare species compared to the former. In addition, the average number of individuals of each species for 100 species is more (five times) than that for 500 species, meaning that the latter SAD has more rare species compared to the former SAD.

**Figure 2:**
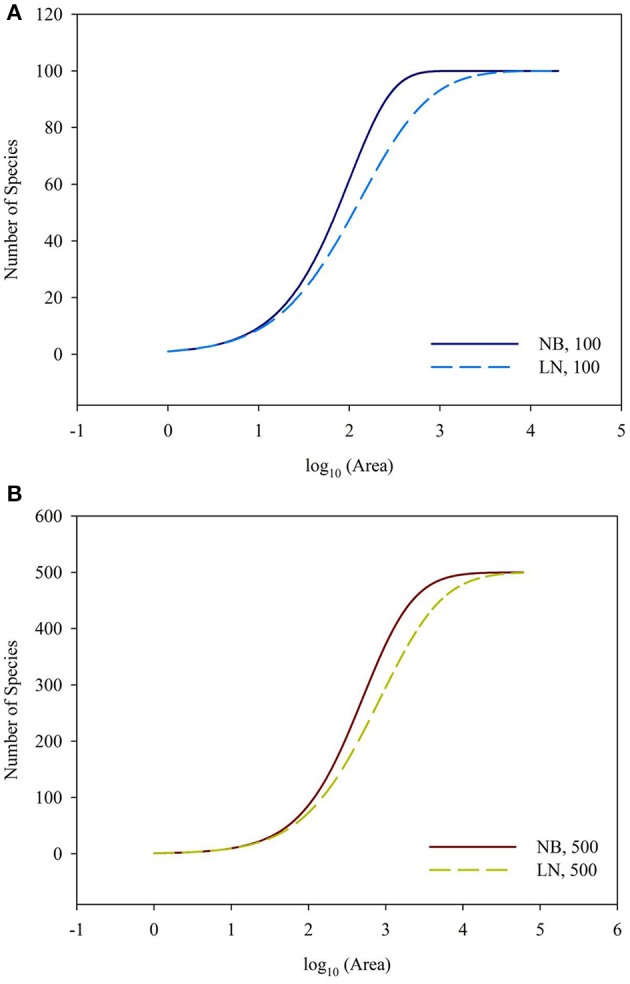
**(A)** Is the diagram for log_10_ (Area)—Number of Species for the number of total species equals 100. **(B)** Is the diagram for log_10_ (Area)—Number of Species for the number of total species equals 100. Different SAD (NB, negative-binomial; LN, log-normal).

**Figure 3:**
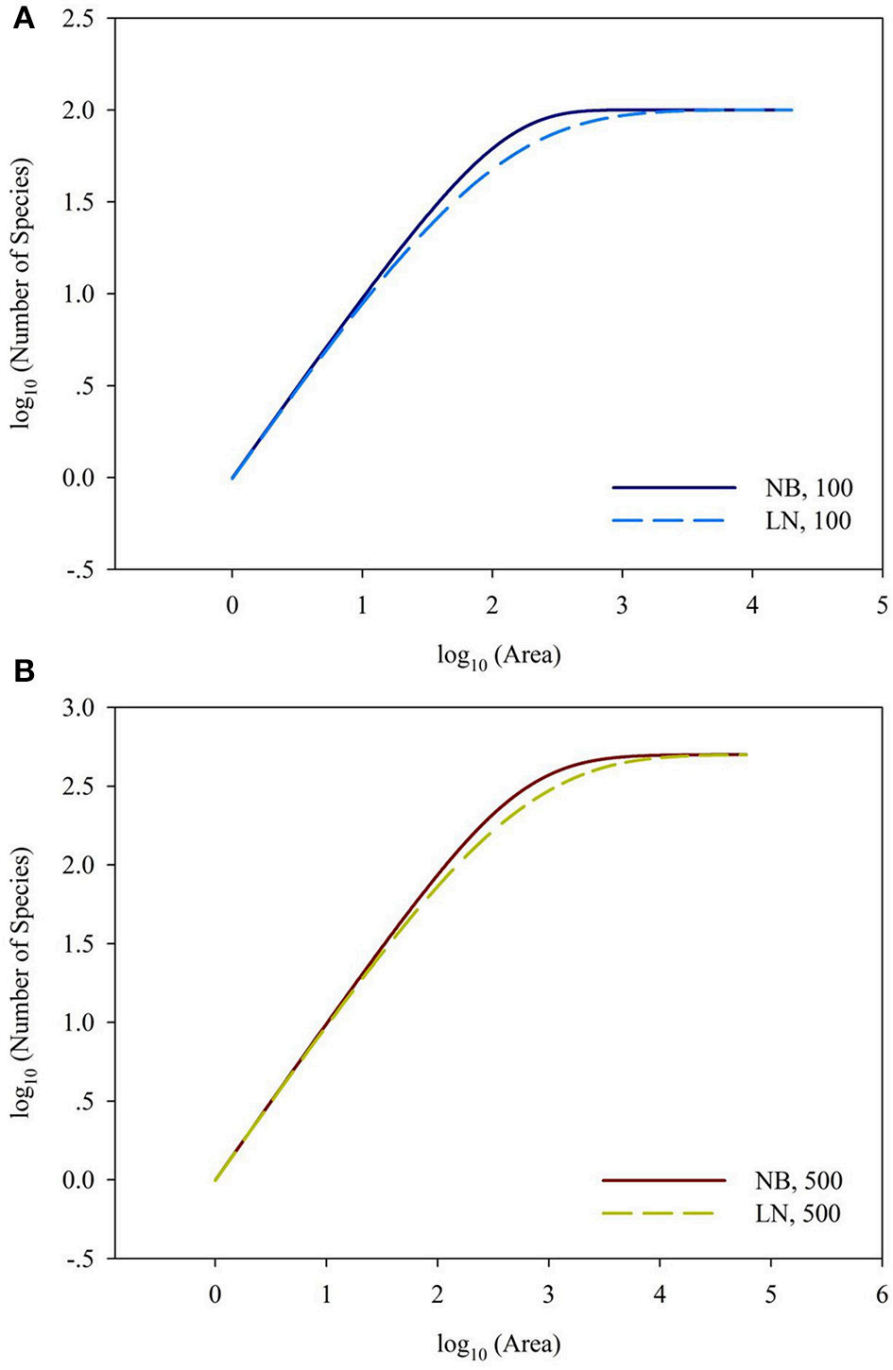
**(A)** Is the diagram for log_10_ (Area)—log_10_ (Number of Species) for the number of total species equals 100. **(B)** Is the diagram for log_10_ (Area)—log_10_ (Number of Species) for the number of total species equals 100. Different SAD (NB, negative-binomial; LN, log-normal).

For the sampling data, the (log-transformed) area was plotted against the number of species (log-transformed or not) in Figures 2, 3. For the log_10_ (Area)−Number of Species, the curves showed downward trend (concave) when the sampling area was small, while the curves shifted to upward trend (convex) when the sampling area reached an inflection point (Figures 2A,B). And it is faster for 100 species to reach the total number of species than that of 500 species. This situation is the same as the NB compared to the LN. For the log_10_ (Area)−log_10_ (Number of Species), the curves showed an upward trend (convex) (Figures 3A,B). Similarly, it is faster for 100 species reach the total number of species than that of 500 species. This situation is the same as the NB compared to the LN. Moreover, the shape of the curves was not largely different for both NB and LN distributions and the total number of species (100 and 500), while the detailed shape of the curves was affected. In summary, the shape of curves was steeper for the NB distribution than for the LN distribution, and the shape of curves was steeper for 100 species than for 500 species.

**Figure 4:**
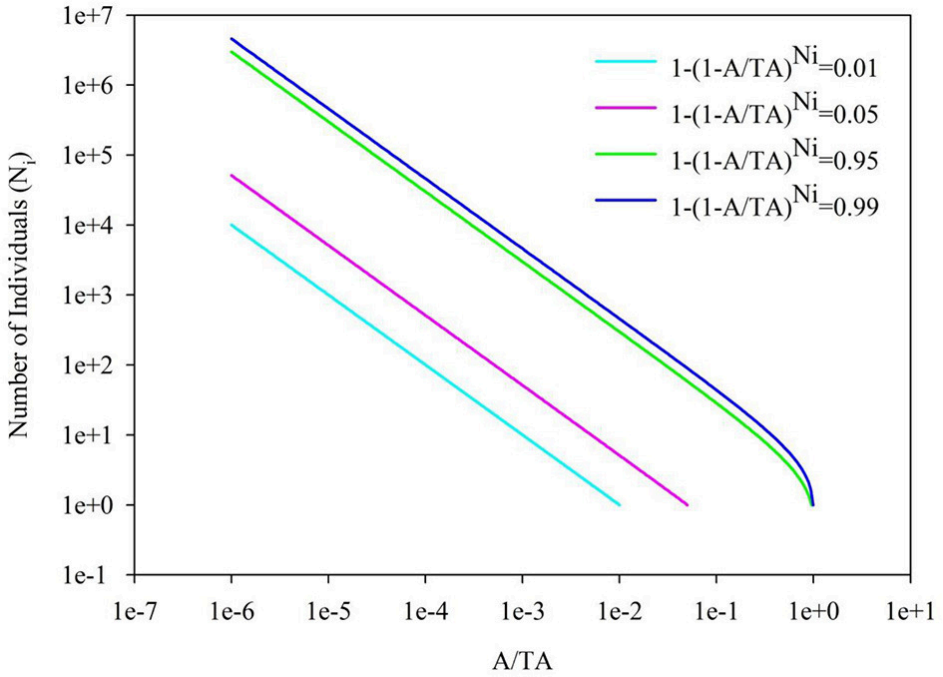
**The diagram for the A/TA−Number of Individuals (*N*_**i**_)**. 1-(A/TA)^*Ni*^ = 0.01, 0.05, 0.95 and 0.99.

Back to the SAR calculated from SAD, the sampling size, the number of individuals per species and their correspondence are important, besides the evenness of SAD. In Figure 4, it showed the 1-(1-A/TA)^*Ni*^ = 0.01, 0.05, 0.95, and 0.99 for the number of individual of a single species. If the sampling area in the space between the 0.01–0.99 lines cyan and blue (or 0.05–0.95 lines pink and green, Section 2 in Figure 4), the number of individuals per species will play a numerical function in the SAR function. These situations also included the transition from the area (Section 1 in Figure 4) below the line cyan (or pink) to the area (Section 2 in Figure 4) above the line cyan (or pink), and the transition from the area (Section 2 in Figure 4) below the line blue (or green) to the area (Section 3 in Figure 4) above the line blue (or green). Obviously, different number of individuals will lead to different additional function in different sampling areas (Tjørve and Tjørve, 2008). If the sampling area is small, only common species have influence on the SAR curve, and rare species are rarely present in the samples due to their low abundance (Preston, 1948; Verberk, 2011); if the sampling area is intermediate, only rare species have influence on the SAR curve, because the value calculated from common species (almost) equals 1; if the sampling area is large, no species has influence on the SAR curve because all species (almost) equals 1. Obviously the parameters of curves (Figures 2, 3) in this study are affected by the SAD of simulated data (Figure 1) and the total number of species.

**Figure 5:**
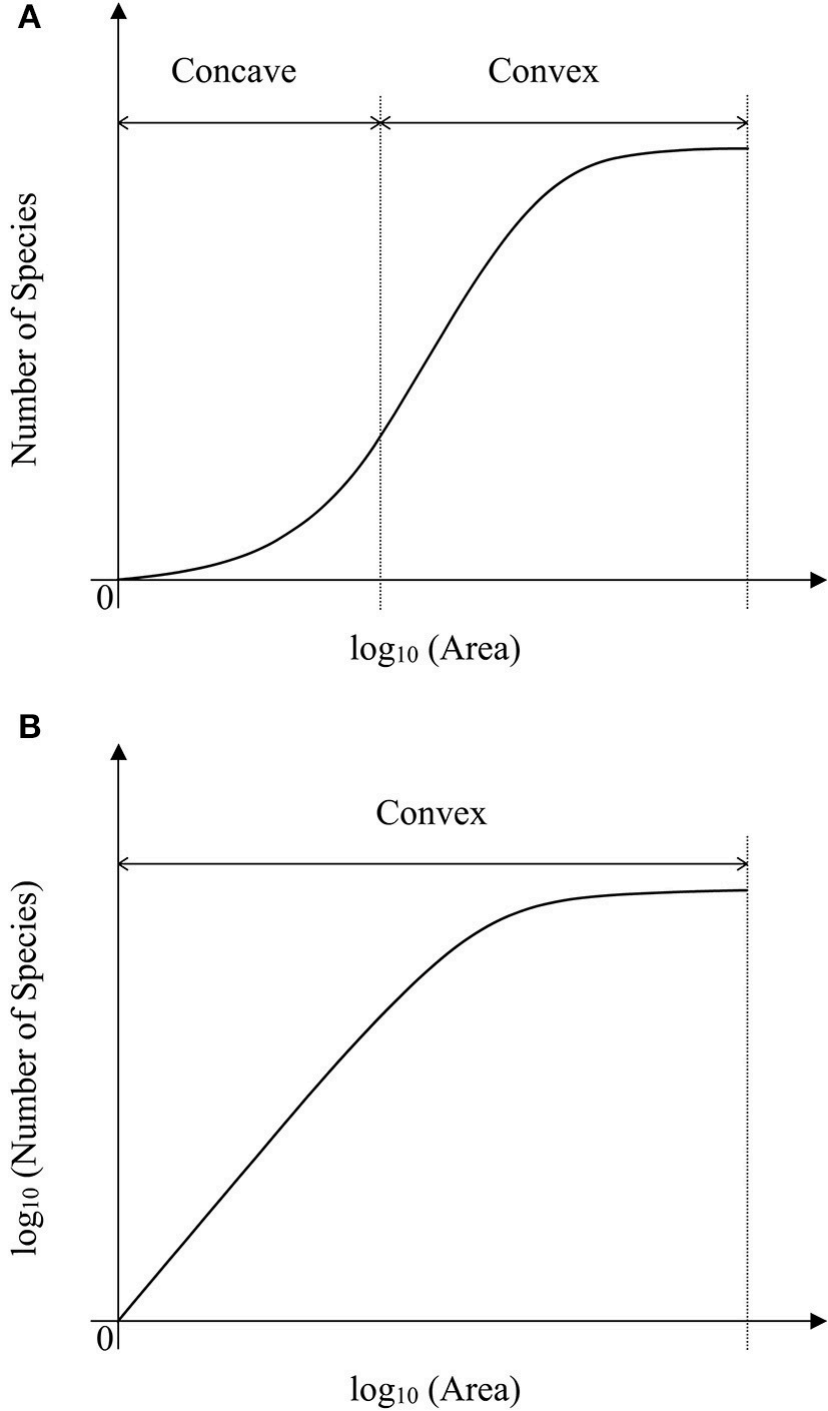
**(A)** Is the schematic diagram for log_10_ (Area)−Number of Species. **(B)** Is the schematic diagram for log_10_ (Area)−log_10_ (Number of Species).

As showed in a generalized schematic diagram of the SARs (Figures 5A,B), species abundance distributions largely affect the shape of SAR curves, which is in accordance with the findings in previous studies (Allen and White, 2003; Green and Ostling, 2003; Šizling and Storch, 2004; Dengler, 2008; Tjørve and Tjørve, 2008; Tjørve et al., 2008; Mokany et al., 2013; Rybicki and Hanski, 2013; Guo, 2015; Harte and Kitzes, 2015). The curve of the logarithm SAR sampling [i.e., log_10_ (Area)–Number of Species] showed a sigmoid shape that can be divided into two sections, concave section when the sampling area is small until the inflection point, and convex section. However, the curve of the power SAR sampling (i.e., log_10_ (Area)–log_10_ (Number of Species)) only has convex section. As shown in Figure 5, the power and logarithm SAR relationship can be linearly well-fitted if the sampling size is not large. And the total number of species is the determinant factor on how height of the plateau will be. The classical SARs were usually fitted to field observations when the sampling size is small or intermediate. It is, however, not practical to scrutinize all the species with accurate numbers in a large area (Pan and Zhu, 2015), and that is the way of extrapolation often used in the literature.

**Figure 6:**
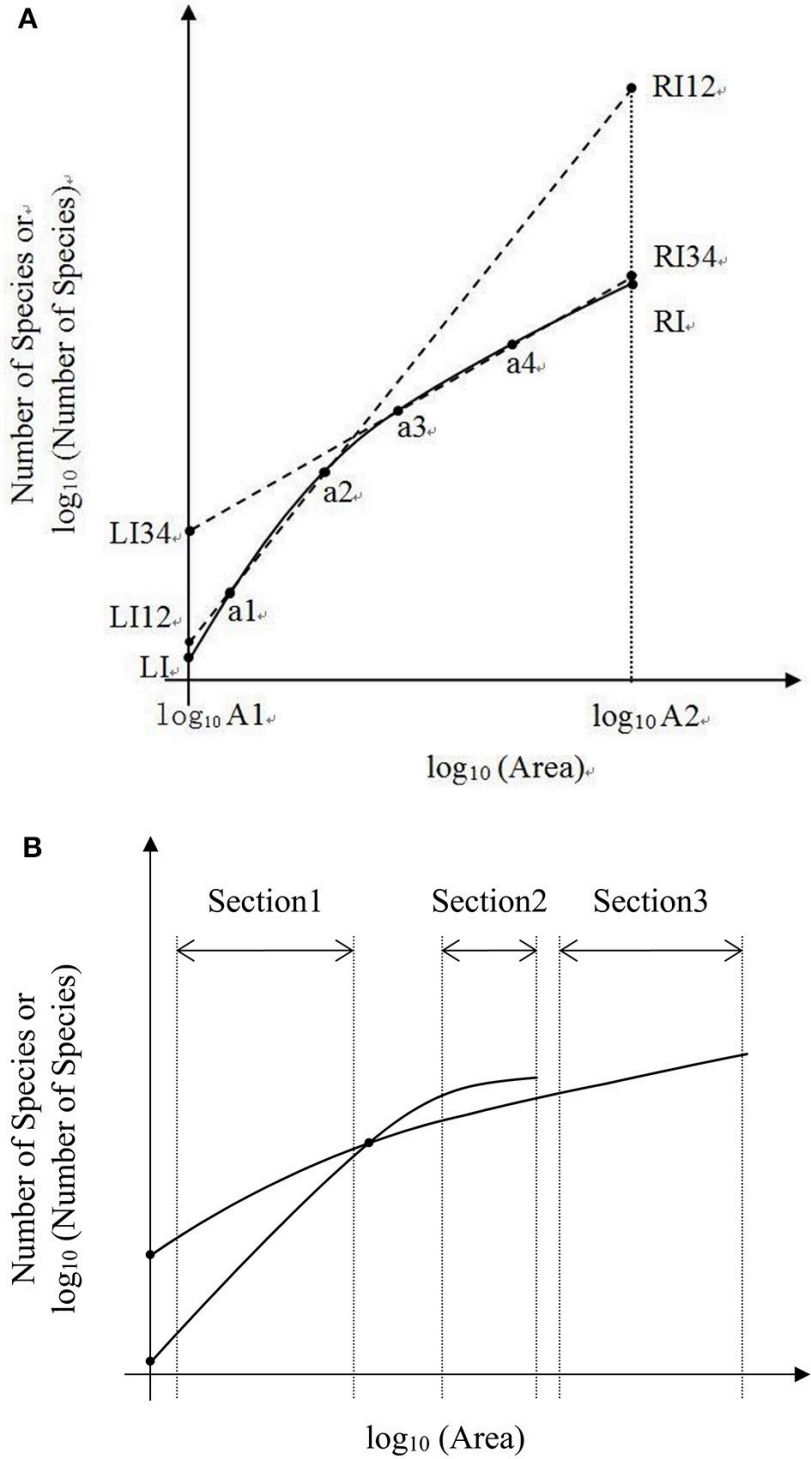
**(A)** Is the schematic diagram of one Species-Area Relationship for log_10_ (Area)−Number of Species or log_10_ (Number of Species). **(B)** Is the schematic diagram of two Species—Area Relationships for log_10_ Area−Number of Species or log_10_ (Number of Species).

In the convex section of a curve, the slope decreases as the area increases, therefore it can lead to overestimate of parameters if one assumes the slope is constant (Figure 6A). For example, the SAR fitting in the small sampling area (a1 and a2) and in the large sampling area (a3 and a4) causes an overestimate of the left intercept (LI) and the right intercept (RI), respectively. Meanwhile, the SAR fitting in the small sampling area has lower LI and higher RI than that in the large sampling area. Overestimate of the number of species would be even higher at the end of a curve. However, in the concave section of an SAR curve, underestimate would occur for the left and right intercepts (Figure 5A), respectively. Therefore, the linear extrapolation of the SAR fitting would be problematic, since the range of the sampling area greatly affects the linearity of the SAR. If the sampling range covers both the concave and convex sections [e.g., in the log_10_ (Area)−Number of Species], misestimate can also occur and would be a little complicated, with one possibility that the left intercept would be underestimated while the right intercept would be overestimated. Thus, the total number of species, derived from the extrapolation from power and logarithm SARs, is not accurate, although this is a very global important parameter for other parameter estimates such as extinction rate (Pan, 2013).

In the convex section of the two SAR curves (Figures 5A,B), the fittings can also be different. Moreover, the estimated parameters (i.e., the slope and intercept) would vary if the sampling areas vary, and even a curve will not exist when the sampling area reached a certain value (Figure 6B). A pattern similar to that in Figure 6B was found in the log-log SAR of Highlands Hammock State Park, Florida, thus there is not necessarily proportionately fewer species loss at broader spatial scales (Powell et al., 2013). This implied that the linear fitting and the comparison of two or more power or logarithm SARs is less problematic only when the sampling area is within the appropriate range. Considering the impact of incomplete surveying and Preston and Pan's effect on the SAD, this SAR comparison will not make any ecological meaning without mathematical endorsement.

In conclusion, neither the power SAR nor the logarithm SAR fitted to simulated data is linear at large sampling range as commonly assumed in previous studies, no matter the distribution of species abundance is log-normal or negative-binomial. Therefore, misestimates can occur if the fitted relationship is extrapolated beyond the range of the small and intermediate sampling sizes. However, if we know the full spatial distribution of all species, we can calculate the SAR curve from SAD, and the sampling and fitting is not useful anymore. Here the dilemma of SAR fitting emerges: you will get the SAR but make mistakes using the sampling and fitting if you do the extrapolation; you can avoid the mistakes using more information, but you do not need sampling and fitting anymore. Obviously, the SAR should be used with caution, as the extrapolation or prediction should not be made if one does not know the whole picture, because the global-local inconsistency exists in SAR (Elith and Leathwick, 2009). In the future, detailed sampling of SAD with full spatial information is the direction, instead of counting the number of species in the area, which also has the Preston and Pan effects in the practice (Pan and Zhu, 2015). For different types of SAR, fitting functions and SAD, such as the island SAR with areas of varying size, whether the linear regression displays global-local consistency deserves more research (Scheiner, 2003).

In addition, the global-local consistency and inconsistency should be given more concerns in ecology. In this study, the community with species distributed randomly and independently is a simplified case, which still has this inconsistency. For the complex ecosystem with inaccurate sampling, spatial-temporal heterogeneity and scale effect, this inconsistency may be more obvious or more unpredictable. For example, the effect of global climate change on different places is different, while one will be hotter, and the other will be drier. In this situation, how to sample to infer the whole picture from limited samples is a challenge for us. A potential method is mass complete survey of one area conducted by an integrated research group/program, rather than three or nine repeated samples per sites.

## Author contributions

XP, FW, and SZ designed the research. XP and XZ carried out the model simulation. XP did the data analysis. XP and FW drafted the manuscript. XP and SZ revised the manuscript.

### Conflict of interest statement

The authors declare that the research was conducted in the absence of any commercial or financial relationships that could be construed as a potential conflict of interest.
